# A Refractive Index Sensor Based on the Resonant Coupling to Cladding Modes in a Fiber Loop

**DOI:** 10.3390/s130911260

**Published:** 2013-08-23

**Authors:** Mauricio Reyes, David Monzón-Hernández, Alejandro Martínez-Ríos, Enrique Silvestre, Antonio Díez, José Luis Cruz, Miguel V. Andrés

**Affiliations:** 1 Departamento de Física Aplicada, Instituto de Ciencia de los Materiales, Universidad de Valencia, Burjassot 46100, Spain; E-Mails: Mauricio.Reyes@uv.es (M.R.); antonio.diez@uv.es (A.D.); jose.l.cruz@uv.es (J.L.C.); miguel.andres@uv.es (M.V.A.); 2 Grupo de Sensores Ópticos y Microdispositivos, Centro de Investigaciones en Óptica A.C., Loma del Bosque 115, col. Lomas del Campestre, Leon, Guanajuato 37150, Mexico; E-Mail: amr6@cio.mx; 3 Departamento de Óptica, Universidad de Valencia, Burjassot 46100, Spain; E-Mail: enrique.silvestre@uv.es

**Keywords:** mode coupling, optical fiber devices, refractive index sensor

## Abstract

We report an easy-to-build, compact, and low-cost optical fiber refractive index sensor. It consists of a single fiber loop whose transmission spectra exhibit a series of notches produced by the resonant coupling between the fundamental mode and the cladding modes in a uniformly bent fiber. The wavelength of the notches, distributed in a wavelength span from 1,400 to 1,700 nm, can be tuned by adjusting the diameter of the fiber loop and are sensitive to refractive index changes of the external medium. Sensitivities of 170 and 800 nm per refractive index unit for water solutions and for the refractive index interval 1.40–1.442, respectively, are demonstrated. We estimate a long range resolution of 3 × 10^−4^ and a short range resolution of 2 × 10^−5^ for water solutions.

## 1. Introduction

Refractometric techniques have experienced a remarkable evolution in the last decade, motivated by the fact that refractive index (RI) measurement gives important information in medical, chemical, industrial and environmental applications. In most modern applications, there is a demand for miniaturization, real time and *in-situ* sensing. Fiber-based technology provides feasible, simple, low cost, and highly sensitive alternatives. Product of this interest, several RI sensors, based on mature fiber technologies, such as highly sensitive long period fiber gratings [1], core-exposed fiber Bragg gratings [2,3], modal all-fiber interferometers [4], or fiber tapers [5,6], have been recently proposed. Furthermore, attractive approaches have been reported taking the new possibilities opened by the advent of the holey fibers [7]. Most fiber-based RI sensors exploit the interaction of the evanescent field with the external medium. Therefore, either polishing, etching, tapering or drilling a section of fiber will be required to prepare a sensor head [1–7]. These post-processing techniques are technically demanding, and make the fiber fragile. In order to overcome this problem, standard long period gratings (LPG) [8], LPG-based interferometers [9] and short multimode fiber interferometers [10], which preserve the integrity of the fiber, can be used.

Here, we propose an easy-to-build and robust optical fiber refractive index sensor, based on the resonant coupling between the fundamental mode of a standard single-mode fiber and the cladding modes, *i.e.*, the high order modes of the fiber itself, in a circular fiber loop. Thus, the fiber integrity is preserved, requiring no Bragg or long period grating, but a simple circular loop of a relatively small radius. However, the polymer coating has to be removed in order to allow the interaction of the cladding modes with the external medium, which reduces in part the robustness of the fiber. The coupling between core and cladding modes is produced by the refractive index profile perturbation that the curvature of the fiber generates. The resonant transfer of power takes place at the wavelengths where the core mode satisfies the phase matching condition with a cladding mode. Therefore the dependence of cladding modes modal index with the external refractive index makes the wavelength of a given resonance to shift. Thus, measuring the wavelength shift of the transmission notches it is possible to determine small refractive index changes of the external medium. Our approach avoids the chemical etching process reported in [11], where a single loop of standard single-mode fiber with a reduced cladding diameter is used. Moreover, we provide an improved theoretical analysis, we estimate the temperature effects and we implement a wavelength codified operation of the sensor, in addition to the simple power loss characterization. Wavelength codified sensors are more reliable than amplitude codified sensors, so this approach is in principle preferable.

## 2. Mode Coupling in Curved Fiber

**Figure 1.**
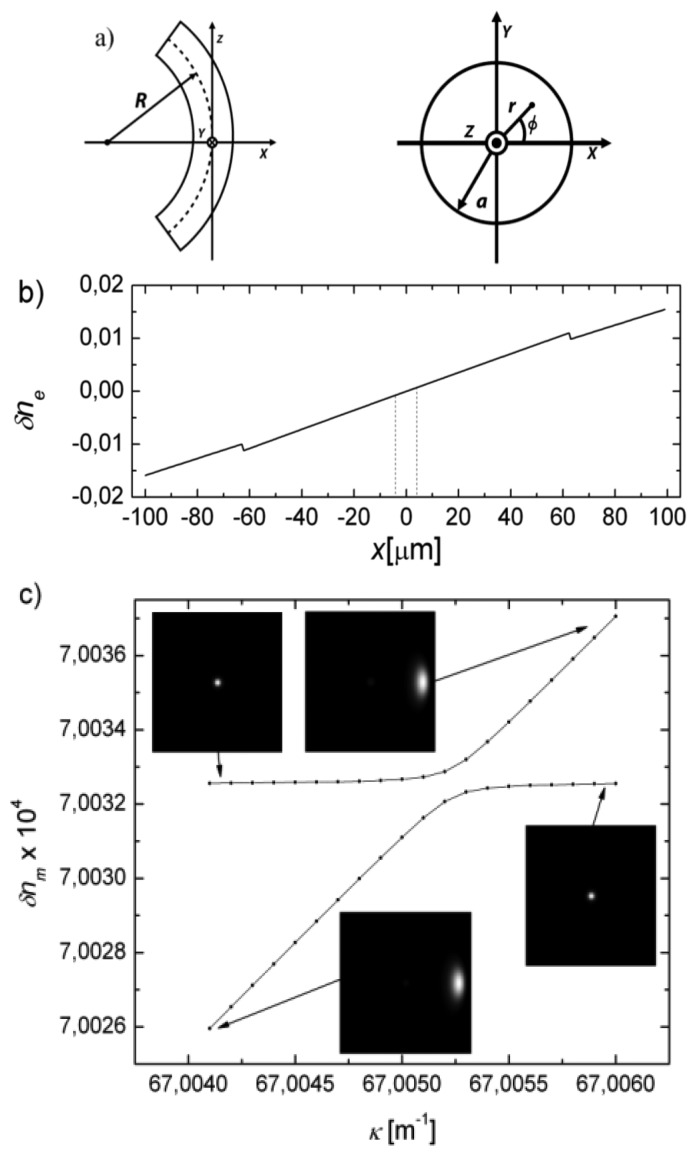
(**a**) Geometry of bent fiber. (**b**) Refractive index perturbation for a bending radius of 6.35 mm; the position of the core is indicated with two dotted lines. (**c**) First resonant coupling predicted by theory as a function of curvature; the insets show the mode intensity patterns of the two coupled modes in a box of 133 × 133 μm.

The strain generated along the fiber, when it is bent, produces anisotropic and asymmetric changes of the dielectric constant due to the photo-elastic effect [12]. In addition, the geometrical effect associated to the curvature of a waveguide can be modeled by an effective refractive index perturbation in an equivalent straight waveguide [13]. The combination of these two effects can be described as a perturbed straight fiber with an effective relative dielectric constant profile:

(1) $$\begin{array}{ll} \varepsilon_{x}(r,\phi )=\varepsilon_{y}(r,\phi )=\frac{\varepsilon (r)(1+2\kappa \;r\cos\phi )}{\left(1-\varepsilon (r)\alpha \kappa \;r\cos\phi \right)} & ,\;r<a\\ \varepsilon_{z}(r,\phi )=\frac{\varepsilon (r)(1+2\kappa \;r\cos\phi )}{\left(1-\varepsilon (r)\beta \kappa \;r\cos\phi \right)} & ,\;r<a\\ \varepsilon_{x}(r,\phi )=\varepsilon_{y}(r,\phi )=\varepsilon_{z}(r,\phi )=\varepsilon_{\text{ext}}(1+2\kappa \;r\cos\phi ) & ,\;r<a \end{array}$$

where *ε*_x_, *ε*_y_ and *ε*_z_ are the effective relative dielectric constants of the perturbed medium in the transverse x and y directions and the longitudinal z direction, respectively; *ε* is the dielectric constant profile of the fiber; *κ* is the curvature of the fiber (*κ* = 1/*R*, *R*: radius of curvature); *r* and *ϕ* are the cylindrical coordinates (*x* = *r* cos*ϕ*), as it is depicted in Figure 1; *α* = *σp*_11_ + (*σ*−1) *p*_12_and *β* = 2 *σp*_12_ − *p*_11_, being *σ* the Poisson's ratio (*σ*= 0.16 for silica) and *p*_ij_ the strain-optic tensor (*p*_11_ = 0.12, *p*_12_ = 0.27, for silica); *ε*_ext_ is the external dielectric constant; and *a* is the cladding radius of the fiber. In the external medium, being either air or a liquid, no strain effect is assumed. A first order approximation of Equation (1) was used in [14] to model bending effects on long period fiber gratings. Other previous theoretical analysis of bending losses in single mode fibers, as those reported in [11,15–17], develop a perturbation method based on a scalar approximation for the electromagnetic fields, assuming a plane interface, and considering no photo-elastic effect, *i.e.*, only the geometrical effect were taken into account. In comparison, here we develop a truly vector modal analysis of the bent fibers, which includes both geometrical and the photo-elastic effects, where the oscillations of reported bend-loss curves are explained in terms of the resonant couplings between the fundamental mode and the cladding modes of the bent fiber.

Figure 1 includes a plot of the effective index perturbation in the x direction, *δn*_e_ = *n*_e_(*r, ϕ*)–*n(r)* when *r* < *a* and *δn*_e_ = *n*_e_(*r, ϕ*)–*n*_ext_ when *r* > *a*, along the x axis, where *n*_e_ = (*ε*_x_)^1/2^ and *n* = (*ε*)^1/2^, computed for a standard SMF28 single-mode fiber (core radius: 4.1 μm, cladding radius: 62.5 μm, numerical aperture: 0.13) with a bending radius of 6.35 mm. In all our simulations we have taken into account the chromatic dispersion of silica (cladding index: 1.444024 at 1,550 nm).

As a result of bending, the modal index of higher order modes increases strongly –while the modal index of the fundamental mode has only a small increase–, and, eventually, when it matches the fundamental mode index a resonant coupling is produced, and efficient power transfer between the fundamental mode and the corresponding cladding mode is enabled. This will be observed as an attenuation dip in the transmission spectrum of the fundamental mode. In order to implement a refractive index sensor, we can take advantage of the natural dependence of the cladding modes modal index with the external refractive index, which will make the spectral position of the transmittance notches to shift as a function of the external refractive index.

In order to calculate the propagation factors of the modes guided by the bent fibers, we followed the method described in [18]. This numerical technique allows fast and accurate modal analysis of special fibers by using an iterative Fourier method. This approach permits dealing with arbitrary spatial refractive-index distributions. The spatial refractive index distribution used for the simulations of the bent fibers corresponds to the effective relative dielectric constant of Equation (1).

In Figure 1c, we can see the first resonant coupling by plotting the modal index deviation, *δn_m_*, with respect the fundamental mode index at *κ* = 0, *λ* = 1,550 nm, as a function of curvature, when the fiber loop is surrounded by air. This coupling corresponds to the original modes LP_01_ and LP_02_. The fields of mode LP_02_ are already strongly distorted by the perturbation. One can follow easily the dispersion curve of the fundamental mode and the higher order cladding mode around the anticrossing point.

As it is shown in Figure 1, the first resonant coupling takes place when the curvature radius is ∼15 mm. This first coupling has a coupling coefficient *k* = 1.6 × 10^−2^ m^−1^, which can be derived from the minimum value of the separation, *δn_m,min_*, between the dispersion curves: *k* =π *δn_m,min_*/*λ* [19]. Such a small coupling coefficient would require a relatively large length of interaction to produce a significant power transfer: about 48 m for 50% power transfer. With the objective to make a practical proposal, we focused our experiments on smaller radius with higher coupling coefficients that may give rise to compact sensor heads. For higher curvatures the cladding modes involved in the experiments will be relatively high order modes. In addition, the curvature enhances the evanescent fields of the cladding modes since the fields are moved towards the external part of the loop (see inset in Figure 1c and, consequently, we expect to achieve higher external refractive index sensitivities by reducing the curvature radius of the fiber.

The modal analysis that we have carried out demonstrates that only at the wavelengths where a phase matching condition is satisfied, as that depicted in detail in Figure 1c a notch in the transmittance is produced, as a result of the distributed transfer of power between the fundamental mode and a perturbed cladding mode along the bent fiber. This type of transfer of power between modes, is what we call resonant coupling. Alternatively, an equivalent modal interferometer description can be used, in which at the point where the curvature starts one should calculate the projection of the fundamental mode onto the perturbed modes of the bent fiber, and after propagation along the length of one loop the modes should be projected back onto the fundamental output mode. We prefer the description in terms of resonant coupling to the modal interferometric approach, due to the modal perspective that we adopt throughout our analysis and because both theory and experiments show that the transfer of power is produced in relatively narrow wavelength bands, while one uses typically a modal interferometer perspective when the transmittance of the system presents a sinusoidal dependence with the wavelength.

## 3. Refractive Index Sensor

**Figure 2.**
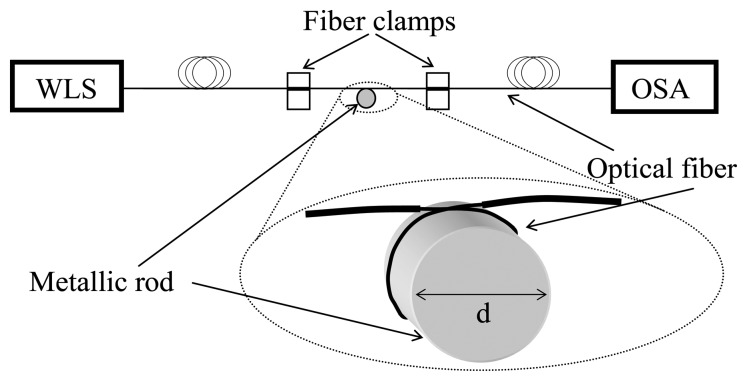
Schematic representation of the experimental set-up. WLS and OSA stand for white light source and optical spectrum analyzer, respectively.

The experimental set-up used to test the proposed RI sensor is shown in Figure 2. The sensor head consists of a small loop of bare optical fiber (SMF-28). The fiber is coiled around a metallic rod with a diameter *d*, which determines the radius of curvature of the fiber, *R* = *d*/2. This metallic rod is a support for the fiber loop, to ensure a constant radius of curvature, but has no influence in the optical response of the device since the fields of the guided modes are pushed towards the outer part of the fiber because of the curvature. All our experiments were carried out using rods with diameters around 12 mm. The polymer coating of the fiber was removed by sinking the fiber during few minutes in a methyl chloride solution, avoiding the use of a mechanical stripper in order to ensure no damage of the fiber surface. The transmission spectrum of the fiber previous to bending was used as a reference signal to normalize the transmission spectra obtained when the fiber loop is formed and immersed in different refractive index solutions.

**Figure 3.**
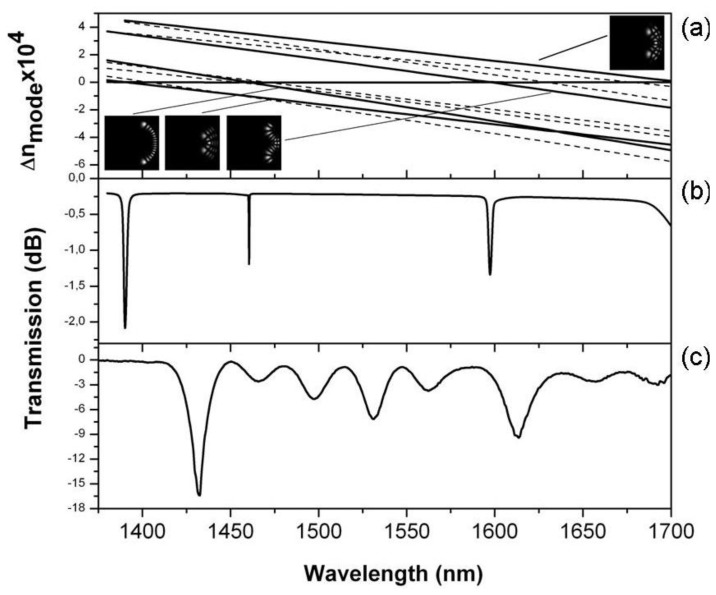
(**a**) High order mode index difference with respect the fundamental mode index with *κ* = 0: coupled modes with anti-crossing curves (solid lines), and non coupled modes (dashed lines). The insets show the mode intensity patterns of the coupled modes in a box of 133 × 133 μm. (**b**) Theoretical transmission spectrum. (**c**) Experimental transmission spectrum. The fiber was wound around a cylinder of 12.70 mm diameter (6.35 mm radius).

Figure 3a gives the calculated high order mode index difference, with respect the fundamental mode index at zero curvature, *versus* wavelength. These values have been computed assuming a radius of curvature of 6.35 mm and air as external medium. The dispersion curves of the modes that exhibit resonant couplings, and give rise to anti-crossing points, are plotted with solid lines, while some other high order modes exhibit no coupling because of the asymmetry of the fields and are plotted with dashed lines. We can observe, in the spectral range 1,375–1,700 nm, four theoretical resonant couplings at 1,390, 1,460, 1,597 and 1,735 nm, with *δn_m,min_* of about 10^−6^, 10^−7^, 10^−6^ and 10^−6^. If we follow the dispersion curves of the modes by reducing the curvature of the fiber down to *κ* = 0, then we can identify the modes of the unperturbed fiber that evolve to the intensity distributions depicted in Figure 3a and give rise to the resonant coupling: LP_24_, LP_81_, LP_62_ and LP_33_, from lower resonant wavelength to higher.

The theoretical transmittance of the fiber loop is shown in Figure 3b. This simulation has been computed projecting the input fundamental mode onto the perturbed modes of the bent section of fiber, allowing the perturbed modes to propagate along the fiber loop with their own propagation factors and projecting the fields onto the output fundamental mode of the fiber at the end of the loop. The spectral position of the notches are determined by the anticrossing points of the dispersion curves, while the minimum transmittance of each dip depends on the accumulated phase difference and the amplitude of excitation of the modes when the projection is computed.

The experimental transmission spectrum of the fiber wound around a cylinder of 12.70 mm diameter is shown in Figure 3c. It is possible to distinguish several peaks, but those with higher losses are located at 1,432 and 1,613 nm. A relative good correspondence between the theoretical analyses and the experimental results can be established if we assume that the experimental wavelengths of the resonant couplings are shifted about 40 nm towards longer wavelengths with respect the theoretical values. Such a shift could be produced by the mechanical tension required to hold the fiber around the metallic rod and the approximations involved in the theoretical simulations. In addition, we have to point out that no artificial adjustment of the nominal values of the fiber has been carried out and that the parameters considered for the simulations are the nominal values depicted in Section 2. Any simulation involving cladding modes, as for example in the case of long period gratings, is highly sensitive to small changes of fiber and material parameters. In our case, no attempt to force a match between theory and experiment has been carried out, since we are more interested in the discussion of the physical mechanism exploited by the sensor proposal.

The difference in amplitude between the theoretical and the experimental notches are probably due to the transient from *κ*= 0 to the curvature determined by the rod, which is not considered in the simulations and that may have a significant effect on the projection of the input field onto the fields of the bent fiber. The shallow dips that the experimental transmission spectrum exhibits between 1,450 and 1,575 nm are produced by smaller couplings to asymmetric higher order modes. Even asymmetric modes that, in principle, should be uncoupled, can give rise to small resonant couplings because of any break of the symmetry in the experiment. Experimentally, a reduction of the radius of curvature of 0.175 mm produces a shift of the transmission dips of 25 nm towards longer wavelengths, showing a strong dependence on the radius of curvature. However, the transmission spectra are highly repeatable; and we have not observed changes in the spectrum every time the fiber was wound around the same cylinder.

According to our theoretical results, the transmission spectrum depicted in Figure 3c should not be interpreted as interference fringes between two modes which phase difference changes as a function of wavelength. In the wavelength range of the experiment we have eight notches (the three stronger notches at 1,432, 1,526 and 1,613 nm and five shallow dips at 1,463, 1,500, 1,562, 1,658 and 1,692 nm) that correspond to the eight cladding modes shown in the theoretical dispersion curves of Figure 3a (three symmetric cladding modes that give rise to the stronger couplings and five asymmetric modes that exhibit weaker couplings). Each cladding mode produces separately a notch in the transmission spectrum, although the relatively large number of couplings generates some overlapping between notches.

In order to analyze the possibility to use the small fiber loop as a refractive index sensor, we measured experimentally the wavelength shift of the resonant couplings as a function of the external refractive index. These measurements were carried out, first, using commercial Cargille liquids with calibrated RI from 1.360 to 1.442 (nominal values at 519 nm and 25 °C), and later using standard solvents as water, acetone and isopropyl alcohol.

**Figure 4.**
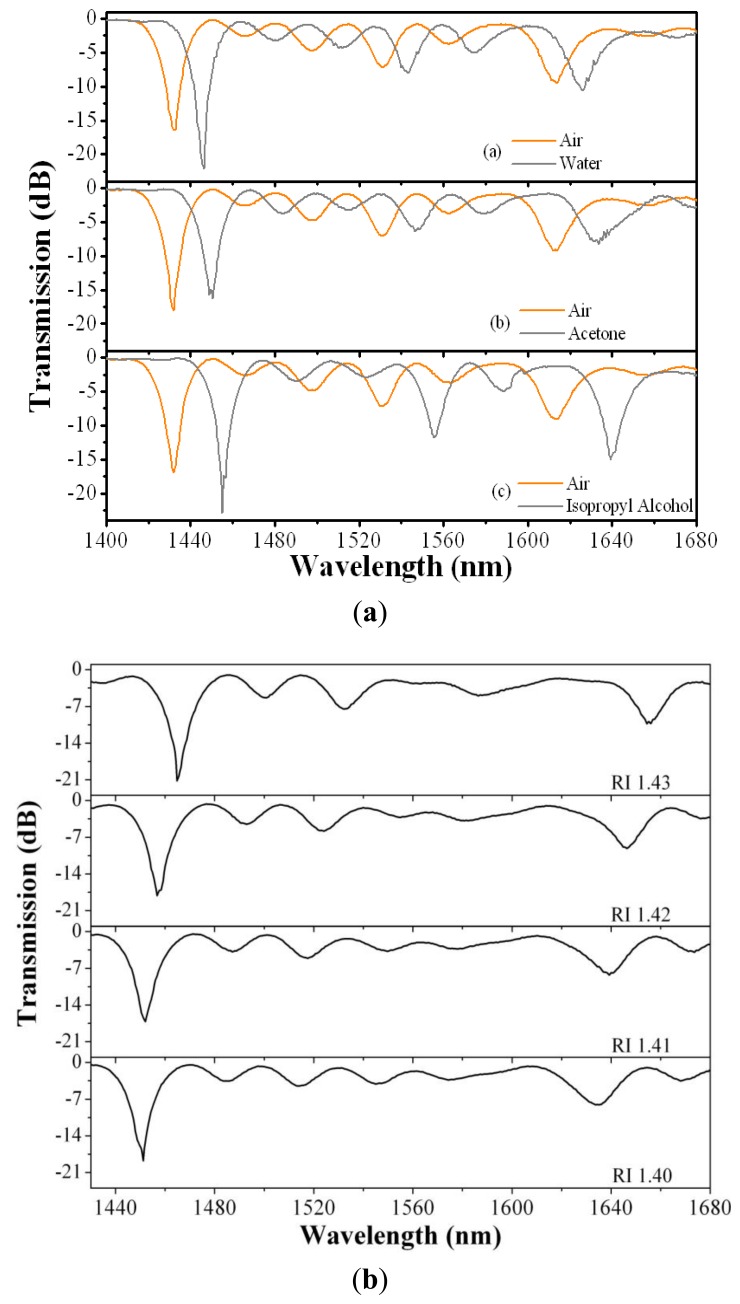
(**a**)Transmission spectra of the sensor for different liquids, when the diameter of the loop was 12.70 mm. (**b**) Transmission spectra of the sensor for different RI liquids. The diameter of the loop was 12.70 mm.

**Figure 5.**
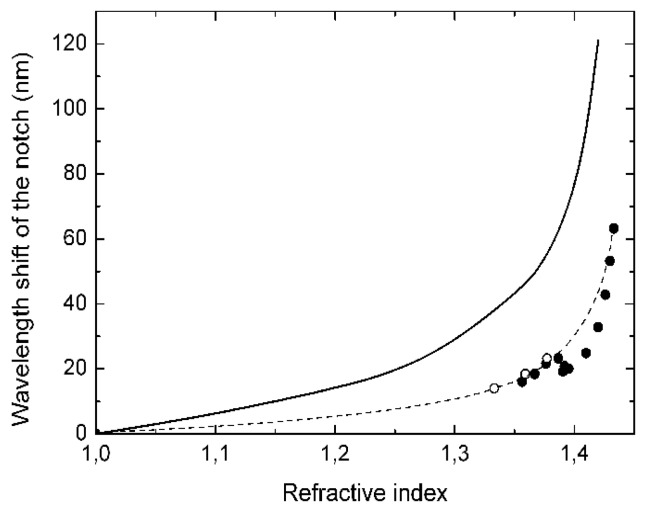
Wavelength shift of a resonant coupling versus external refractive index: (solid line) theoretical shift of the resonance, (solid circles) experimental values obtained with Cargille liquids, (open circles) experimental values obtained with standard liquids (see Figure 4), (dashed line) eye guide.

Between consecutive measurements, the fiber loop was cleanedwith acetone and then dried with air. As an example, Figure 4a gives the transmission spectra whenwater, acetone and isopropyl alcohol were applied, while in Figure 4b we show the transmission spectra for four different RI liquids. The wavelength ofeach resonant coupling shift towards longer values as the refractive index of the external medium augments. A more detailed calibration is given in Figure 5 for the strongest experimental coupling, the one located at 1,432 nm when the fiber is surrounded by air.

The theory predicts correctly the tendency, although it fails to give the exact values for the wavelength shifts. In addition, the experimental points show an anomalous behavior around 1.39. That anomaly is likely produced by a resonant coupling between three modes. The simultaneous phase matching of three modes has been observed in the numerical simulations around that point, between two coupled modes and, in principle, one uncoupled mode. However, as we have mentioned already, the experiment shows that it exists a break of the symmetry and most of the high order modes are slightly coupled. Thus, around 1.39 we have a three modes coupling that will give rise to a complex anticrossing point in which the gap between the dispersion curves of the modes has a non trivial dependence on the wavelength. We think that this is the reason for the somewhat anomalous experimental behavior. Nevertheless, around the refractive index of water solutions (∼1.33)–—where most practical applications are–—the response is monotonic and has a sensitivity of 170 nm per refractive index unit (RIU). This sensitivity is higher than the values reported for standard LPG which are typically around or below 50 nm/RIU for water solutions [20], as well as higher than the values reported in the references discussed in the introduction. Using chemically etched LPGs, the sensitivity can be improved and a sensitivity of 262 nm/RIU has been reported [21]. Thus, we can estimate a detection limit of 3 × 10^−4^ for water solutions, assuming an OSA resolution of 50 pm. However, this estimation might be somewhat unrealistic since the line width of the dips is about 10 nm. Thus if we assume that we can detect 1/20 of the line width, *i.e.*, a shift of 500 pm, then the estimation would be a detection limit of 3 × 10^−3^. At higher refractive index values, around 1.41, the sensitivity increases to 800 nm/RIU, which gives a detection limit between 6 × 10^−4^ and 6 × 10^−5^. Although these values are relatively low, they are better than that of some commercial bulk refractometers, and similar to that obtained in [11] by etching the fiber diameter to 81 μm. However, our proposal is a wavelength codified sensor which is intrinsically more reliable than amplitude codified sensors as that reported in [11].

Temperature effects are an important issue for any refractive index sensor. A detailed simulation has been carried out in which the thermal expansion of both the core and cladding radii have been taken into account (*α_T_* = 0.55 × 1^−6^ K^−1^), as well as the thermo-optic coefficients of the Ge-doped core (∂*n_core_*/*∂T* = 1.15 × 10^−5^ K^−1^) and the pure silica cladding (*∂n_clad_*/*∂T* = 1.06 × 10^−5^ K^−1^). The theoretical estimation is a drift of 0.1 nm/K, towards shorter wavelengths, for the resonant couplings shown in Figure 3. Such a small drift is comparable to the resolution, so it can be considered negligible provided the measurements are carried out maintaining a constant temperature within ±1 K.

Moreover, if we decided to operate our sensor as an amplitude codified sensor by measuring transmittance changes *versus* external refractive index sensor, then a detection limit of 2 × 10^−5^ is estimated, since the slope at the optimum point of the transmission spectrum for water is 2.9 dB/nm and a resolution of 0.01 dB is usual in commercial optical power meters. Similarly, a detection limit of 4 × 10^−6^ is obtained for an external refractive index of about 1.41. Thus, combining wavelength and power measurements our proposal exhibits large refractive index range with moderate resolution and short ranges around a given refractive index value with high resolution.

## 4. Conclusions

We have demonstrated an easy-to-build optical fiber refractometer that consists of a single mode fiber wound around a cylinder to form a single loop. The theoretical principle of operation has been discussed and, according to the experimental results, a long range resolution of 3 × 10^−4^ for water solutions is demonstrated, by measuring wavelength changes of the spectral transmission notches. Amplitude measurements, *i.e.*, optical power transmission measurements, would give a resolution of 2 × 10^−5^. At higher refractive index values, the detection limits can be as low as 4 × 10^−6^.
